# Acetato­aqua­{4,4′,6,6′-tetra-*tert*-butyl-2,2′-[(2-pyridyl­meth­yl)imino­dimethyl­ene]diphenolato}manganese(III) ethanol solvate

**DOI:** 10.1107/S1600536810021288

**Published:** 2010-06-16

**Authors:** Elliott Chard, Louise N. Dawe, Christopher M. Kozak

**Affiliations:** a Department of Chemistry, Memorial University of Newfoundland, St John’s, NL, Canada A1B 3X7; b C-CART X-Ray Diffraction Laboratory, Department of Chemistry, Memorial University of Newfoundland, St John’s, NL, Canada A1B 3X7

## Abstract

In the title complex, [Mn(C_36_H_50_N_2_O_2_)(CH_3_COO)(H_2_O)]·CH_3_CH_2_OH, the Mn^III^ atom is in an octa­hedral environment and is coordinated by the tetra­dentate amine–bis­(phenolate) ligand, a monodentate acetate anion and a water mol­ecule. An ethanol solvent mol­ecule is also found in the asymmetric unit. The structure displays O—H⋯O and C—H⋯O hydrogen bonding.

## Related literature

For a related structure, see: van Gorkum *et al.* (2008 ▶). For the structure of the unmetallated ligand, see: Chmura *et al.* (2006 ▶). For synthetic procedures, see: Kerton *et al.* (2008 ▶); Shimazaki *et al.* (2000 ▶).
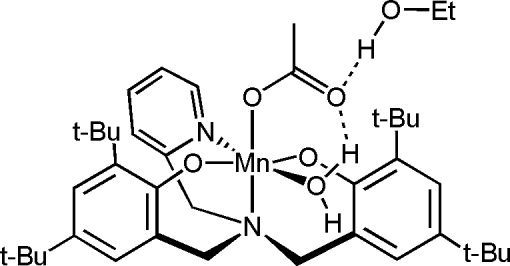


## Experimental

### 

#### Crystal data

[Mn(C_36_H_50_N_2_O_2_)(C_2_H_3_O_2_)(H_2_O)]·C_2_H_6_O
*M* *_r_* = 720.87
Monoclinic, 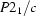

*a* = 16.505 (3) Å
*b* = 10.8310 (16) Å
*c* = 26.512 (5) Å
β = 118.798 (3)°
*V* = 4153.2 (12) Å^3^
*Z* = 4
Mo *K*α radiation
μ = 0.36 mm^−1^
*T* = 153 K
0.40 × 0.30 × 0.24 mm

#### Data collection

Rigaku Saturn diffractometer
Absorption correction: numerical (*ABSCOR*; Higashi, 2000 ▶) *T*_min_ = 0.906, *T*_max_ = 0.948
44205 measured reflections
8589 independent reflections
8248 reflections with *I* > 2σ(*I*)
*R*_int_ = 0.032

#### Refinement

*R*[*F* ^2^ > 2σ(*F* ^2^)] = 0.050
*wR*(*F* ^2^) = 0.138
*S* = 1.10
8589 reflections
444 parameters
H-atom parameters constrained
Δρ_max_ = 0.59 e Å^−3^
Δρ_min_ = −0.50 e Å^−3^

### 

Data collection: *CrystalClear* (Rigaku, 2005 ▶); cell refinement: *CrystalClear*; data reduction: *CrystalClear*; program(s) used to solve structure: *SIR92* (Altomare *et al.*, 1994 ▶); program(s) used to refine structure: *SHELXL97* (Sheldrick, 2008 ▶); molecular graphics: *CrystalStructure* (Rigaku/MSC, 2005 ▶); software used to prepare material for publication: *CrystalStructure*.

## Supplementary Material

Crystal structure: contains datablocks I, global. DOI: 10.1107/S1600536810021288/pv2284sup1.cif

Structure factors: contains datablocks I. DOI: 10.1107/S1600536810021288/pv2284Isup2.hkl

Additional supplementary materials:  crystallographic information; 3D view; checkCIF report

## Figures and Tables

**Table 1:** Hydrogen-bond geometry (Å, °)

*D*—H⋯*A*	*D*—H	H⋯*A*	*D*⋯*A*	*D*—H⋯*A*
O5—H41*A*⋯O6^i^	0.92	1.91	2.799 (2)	160
O6—H42⋯O4^ii^	0.91	1.81	2.723 (3)	171
O5—H41*B*⋯O4	0.93	1.79	2.677 (2)	160
C4—H4*B*⋯O1	0.98	2.36	2.991 (4)	122
C5—H5*C*⋯O1	0.98	2.28	2.929 (3)	123
C15—H15*B*⋯O5	0.99	2.54	3.202 (3)	124
C34—H34*C*⋯O2	0.98	2.45	3.102 (3)	123
C35—H35*A*⋯O2	0.98	2.32	3.010 (3)	126

Symmetry codes: (i) 
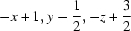
; (ii) 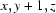.

**Fig. 1.**
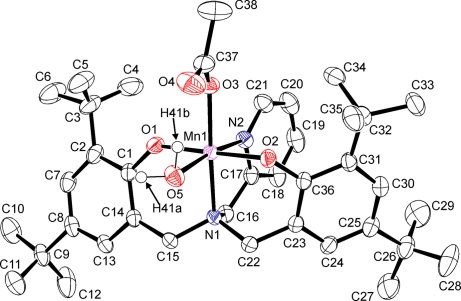
Molecular structure of 1 with atom numbering scheme. Ellipsoids drawn at the 50% probability level. Only the H-atoms on the water ligand (H 41a and H41b) are shown. Ethanol solvate molecule removed for clarity.

**Fig. 2.**
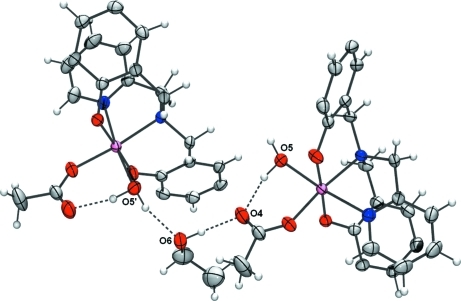
Molecular structure of 1 showing intermolecular hydrogen-bonded linear chain. Ellipsoids drawn at the 50% probability level and t-butyl groups on phenolate rings removed for clarity.

## supplementary crystallographic information

### Comment

In the complex acetato[2-pyridylamino-*N*,*N*-bis(2-methylene-4,6-*tert*-butylphenolato)]aquamanganese(III) (1), the manganese(III) ion resides in a distorted octahedral geometry. Its coordination sphere is occupied by two phenolate oxygen atoms, a tertiary amine and a pyridine nitrogen donor. The phenolate oxygens, O1 and O2, of the tetradentate ligand are *trans* orientated and exhibit Mn1—O1 and Mn1—O2 bond lengths of 1.8532 (14) and 1.8770 (14) Å, respectively. The O3 atom of the monodentate acetate group is *trans* to the amine nitrogen, N1, and the Mn1—O3 and Mn1—N1 bond distances are 1.9958 (15) and 2.1058 (16) Å, respectively. The water ligand, O5, is *trans* to the pyridine nitrogen donor, N2. The Mn ion is Jahn-Teller distorted along the Mn1—N2 and Mn1—O5 bonds, which are considerably elongated giving bond distances of 2.2543 (17) and 2.2434 (15) Å, respectively. This elongated axis is similar to that reported by Van Gorkum *et al.* (2008), except that the previously reported structure possesses a bidentate acetate and no water ligand. Bond angles of *trans*-orientated ligands around Mn are slightly bent and range between 170.77 (6)° for O3—Mn1—N1 and 177.70 (6)° for O1—Mn1—O2.

The complex exhibits both intra- and intermolecular hydrogen bonding. The water ligand acts as a hydrogen bond donor to the uncoordinated O4 of the acetate ligand. The O4···O5 interatomic distance is 2.677 (2) Å and within the typical range for hydrogen bonding between oxygen-containing hydrogen bond donor-acceptors. The uncoordinated O4 of the acetate group also displays intermolecular hydrogen bonding to an ethanol solvate molecule. The interatomic distance between O4 of the acetate and O6 [x, y-1, z] of the ethanol molecule is 2.723 (2) Å, which is within the sum of the van der Waals radii. The ethanol ligand is the hydrogen bond donor and the acetate O6 is the acceptor. Another intermolecular hydrogen bond exists between the coordinated water ligand and the ethanol oxygen atom of a second ethanol molecule (2.7989 (18) Å for O5···O6 [-x+1, y-1/2, -z+3/2]). In this interaction, the water ligand acts as hydrogen bond donor and the solvate ethanol O-atom is the acceptor. These multiple intermolecular hydrogen bonding interactions effectively result in chains where the six-coordinate Mn complexes are bridged by solvate ethanol molecules.

### Experimental

The 2-pyridylamino-*N,N*-bis(2-methylene-4,6-tert-butylphenol) ligand (abbreviated H_2_(O_2_NN')) was prepared according to the published methods (Kerton *et al.*, 2008; Shimazaki *et al.*, 2000). Mn(OAc)_2_.4H_2_O (0.5033 g, 2.0 mmol) and H_2_(O_2_NN') (0.5619 g, 1.00 mmol) were dissolved in 95% ethanol (20 ml) and heated to reflux for one hour. The resulting dark purple solution was filtered through a glass frit while hot. Distilled H_2_O (10 ml) was added to the dark purple filtrate. As the solution cooled to room temperature, dark purple crystals suitable for X-ray diffraction precipitated out of the ethanol/water medium (580 mg, 86% yield). MS (MALDI-TOF) m/z, intensity (ion): 656.3305, 20% ([M—H_2_O]^+^); 596.3156, 42% ([M—H_2_O-OAc]^+^).

### Refinement

H(41 A, 41B) and H(42) were located in difference map positions, and refined on a riding model. All other hydrogen atoms were introduced in calculated positions with distances C—H = 0.95, 0.98 and 0.99 Å for aryl, methyl and methylene type H-atoms and refined on a riding model. Isotropic thermal parameters 1.2 times that of their bonding partners were allowed for all H-atoms.

### Crystal data

**Table:**

[Mn(C_36_H_50_N_2_O_2_)(C_2_H_3_O_2_)(H_2_O)]·C_2_H_6_O	*F*(000) = 1552
*M**_r_* = 720.87	*D*_x_ = 1.153 Mg m^−^^3^
Monoclinic, *P*2_1_/*c*	Mo *K*α radiation, λ = 0.71075 Å
Hall symbol: -P 2ybc	Cell parameters from 15117 reflections
*a* = 16.505 (3) Å	θ = 2.0–30.7°
*b* = 10.8310 (16) Å	µ = 0.36 mm^−^^1^
*c* = 26.512 (5) Å	*T* = 153 K
β = 118.798 (3)°	Irregular, purple
*V* = 4153.2 (12) Å^3^	0.40 × 0.30 × 0.24 mm
*Z* = 4	

### Data collection

**Table:**

Rigaku Saturn diffractometer	8589 independent reflections
Radiation source: fine-focus sealed tube	8248 reflections with *I* > 2σ(*I*)
graphite - Rigaku SHINE	*R*_int_ = 0.032
Detector resolution: 14.63 pixels mm^-1^	θ_max_ = 26.5°, θ_min_ = 2.7°
ω scans	*h* = −20→20
Absorption correction: numerical (*ABSCOR*; Higashi, 2000)	*k* = −13→13
*T*_min_ = 0.906, *T*_max_ = 0.948	*l* = −33→33
44205 measured reflections	

### Refinement

**Table:**

Refinement on *F*^2^	Primary atom site location: structure-invariant direct methods
Least-squares matrix: full	Secondary atom site location: difference Fourier map
*R*[*F*^2^ > 2σ(*F*^2^)] = 0.050	Hydrogen site location: inferred from neighbouring sites
*wR*(*F*^2^) = 0.138	H-atom parameters constrained
*S* = 1.10	*w* = 1/[σ^2^(*F*_o_^2^) + (0.069*P*)^2^ + 2.8924*P*] where *P* = (*F*_o_^2^ + 2*F*_c_^2^)/3
8589 reflections	(Δ/σ)_max_ = 0.001
444 parameters	Δρ_max_ = 0.59 e Å^−^^3^
0 restraints	Δρ_min_ = −0.50 e Å^−^^3^

### Special details

**Table:**

Geometry. All esds (except the esd in the dihedral angle between two l.s. planes) are estimated using the full covariance matrix. The cell esds are taken into account individually in the estimation of esds in distances, angles and torsion angles; correlations between esds in cell parameters are only used when they are defined by crystal symmetry. An approximate (isotropic) treatment of cell esds is used for estimating esds involving l.s. planes.
Refinement. Refinement of *F*^2^ against ALL reflections. The weighted *R*-factor wR and goodness of fit *S* are based on *F*^2^, conventional *R*-factors *R* are based on *F*, with *F* set to zero for negative *F*^2^. The threshold expression of *F*^2^ > σ(*F*^2^) is used only for calculating *R*-factors(gt) etc. and is not relevant to the choice of reflections for refinement. *R*-factors based on *F*^2^ are statistically about twice as large as those based on *F*, and *R*- factors based on ALL data will be even larger.

### Fractional atomic coordinates and isotropic or equivalent isotropic displacement parameters (Å^2^)

**Table:**

	*x*	*y*	*z*	*U*_iso_*/*U*_eq_	
Mn1	0.569186 (18)	0.22108 (3)	0.887302 (11)	0.02241 (10)	
O1	0.44186 (9)	0.23550 (13)	0.85424 (7)	0.0321 (3)	
O2	0.69749 (9)	0.20020 (12)	0.91944 (6)	0.0268 (3)	
O3	0.55745 (10)	0.04484 (13)	0.90481 (6)	0.0328 (3)	
O4	0.57756 (14)	−0.05365 (15)	0.83781 (8)	0.0495 (4)	
O5	0.55651 (10)	0.18014 (14)	0.80084 (6)	0.0334 (3)	
H41A	0.4974	0.1796	0.7701	0.041*	
H41B	0.5646	0.0956	0.8061	0.041*	
O6	0.60773 (12)	0.72486 (15)	0.80117 (7)	0.0424 (4)	
H42	0.6016	0.8027	0.8123	0.051*	
N1	0.57820 (10)	0.41364 (14)	0.88018 (6)	0.0243 (3)	
N2	0.58705 (12)	0.28484 (16)	0.97301 (7)	0.0288 (4)	
C1	0.38156 (13)	0.32682 (18)	0.82789 (8)	0.0271 (4)	
C2	0.28873 (14)	0.31164 (19)	0.81628 (9)	0.0325 (4)	
C3	0.25997 (16)	0.1931 (2)	0.83644 (12)	0.0440 (6)	
C4	0.3141 (2)	0.1835 (3)	0.90203 (13)	0.0579 (7)	
H4A	0.3029	0.2574	0.9191	0.070*	
H4B	0.3802	0.1767	0.9145	0.070*	
H4C	0.2937	0.1102	0.9146	0.070*	
C5	0.2776 (2)	0.0777 (2)	0.80967 (15)	0.0582 (7)	
H5A	0.2429	0.0839	0.7676	0.070*	
H5B	0.2574	0.0042	0.8222	0.070*	
H5C	0.3438	0.0711	0.8221	0.070*	
C6	0.15607 (19)	0.1929 (3)	0.81807 (17)	0.0650 (9)	
H6A	0.1413	0.2656	0.8341	0.078*	
H6B	0.1409	0.1179	0.8325	0.078*	
H6C	0.1200	0.1949	0.7760	0.078*	
C7	0.22611 (14)	0.4063 (2)	0.78625 (9)	0.0342 (4)	
H7	0.1636	0.3966	0.7778	0.041*	
C8	0.25052 (14)	0.5142 (2)	0.76797 (8)	0.0327 (4)	
C9	0.18036 (15)	0.6155 (2)	0.73282 (9)	0.0392 (5)	
C10	0.08308 (18)	0.5871 (3)	0.72413 (12)	0.0579 (7)	
H10A	0.0626	0.5065	0.7054	0.070*	
H10B	0.0399	0.6511	0.6999	0.070*	
H10C	0.0848	0.5856	0.7616	0.070*	
C11	0.17281 (18)	0.6194 (3)	0.67266 (11)	0.0511 (6)	
H11A	0.1527	0.5385	0.6542	0.061*	
H11B	0.2333	0.6397	0.6763	0.061*	
H11C	0.1276	0.6823	0.6492	0.061*	
C12	0.2115 (2)	0.7395 (3)	0.76157 (14)	0.0730 (11)	
H12A	0.2734	0.7578	0.7672	0.088*	
H12B	0.2130	0.7380	0.7990	0.088*	
H12C	0.1682	0.8034	0.7372	0.088*	
C13	0.34281 (14)	0.52737 (19)	0.78144 (8)	0.0299 (4)	
H13	0.3617	0.6006	0.7703	0.036*	
C14	0.40805 (13)	0.43584 (18)	0.81089 (8)	0.0264 (4)	
C15	0.50525 (12)	0.45405 (18)	0.82128 (8)	0.0269 (4)	
H15A	0.5145	0.5426	0.8162	0.032*	
H15B	0.5132	0.4071	0.7919	0.032*	
C16	0.56725 (14)	0.48099 (18)	0.92526 (8)	0.0294 (4)	
H16A	0.5015	0.5040	0.9098	0.035*	
H16B	0.6038	0.5582	0.9348	0.035*	
C17	0.59771 (13)	0.40683 (19)	0.97957 (8)	0.0295 (4)	
C18	0.62907 (16)	0.4634 (2)	1.03279 (9)	0.0414 (5)	
H18	0.6371	0.5504	1.0367	0.050*	
C19	0.64830 (17)	0.3901 (3)	1.08012 (10)	0.0478 (6)	
H19	0.6702	0.4262	1.1171	0.057*	
C20	0.63538 (17)	0.2641 (3)	1.07310 (10)	0.0449 (6)	
H20	0.6471	0.2125	1.1049	0.054*	
C21	0.60499 (16)	0.2145 (2)	1.01892 (9)	0.0367 (5)	
H21	0.5965	0.1277	1.0140	0.044*	
C22	0.67118 (13)	0.44181 (19)	0.88544 (9)	0.0308 (4)	
H22A	0.6774	0.3970	0.8549	0.037*	
H22B	0.6751	0.5313	0.8794	0.037*	
C23	0.74940 (13)	0.40598 (19)	0.94303 (8)	0.0289 (4)	
C24	0.80855 (14)	0.4939 (2)	0.98171 (9)	0.0339 (4)	
H24	0.7978	0.5791	0.9723	0.041*	
C25	0.88294 (14)	0.4583 (2)	1.03370 (9)	0.0345 (4)	
C26	0.94866 (16)	0.5507 (2)	1.07875 (11)	0.0437 (5)	
C27	0.9188 (2)	0.6838 (3)	1.06142 (15)	0.0642 (8)	
H27A	0.8558	0.6952	1.0554	0.077*	
H27B	0.9201	0.7025	1.0257	0.077*	
H27C	0.9610	0.7393	1.0920	0.077*	
C28	1.04710 (18)	0.5335 (3)	1.08810 (15)	0.0664 (9)	
H28A	1.0666	0.4478	1.0992	0.080*	
H28B	1.0892	0.5891	1.1187	0.080*	
H28C	1.0484	0.5527	1.0524	0.080*	
C29	0.9485 (2)	0.5255 (3)	1.13607 (13)	0.0650 (8)	
H29A	0.9677	0.4401	1.1481	0.078*	
H29B	0.8861	0.5384	1.1308	0.078*	
H29C	0.9916	0.5821	1.1657	0.078*	
C30	0.89708 (13)	0.3316 (2)	1.04407 (9)	0.0328 (4)	
H30	0.9497	0.3061	1.0786	0.039*	
C31	0.83970 (13)	0.24007 (19)	1.00753 (8)	0.0283 (4)	
C32	0.86199 (14)	0.1024 (2)	1.02091 (9)	0.0322 (4)	
C33	0.95429 (16)	0.0820 (2)	1.07562 (10)	0.0445 (5)	
H33A	0.9521	0.1204	1.1084	0.053*	
H33B	1.0043	0.1193	1.0708	0.053*	
H33C	0.9656	−0.0068	1.0826	0.053*	
C34	0.78670 (16)	0.0385 (2)	1.02959 (11)	0.0430 (5)	
H34A	0.7825	0.0778	1.0615	0.052*	
H34B	0.8024	−0.0490	1.0384	0.052*	
H34C	0.7272	0.0457	0.9943	0.052*	
C35	0.86841 (16)	0.0416 (2)	0.97090 (10)	0.0406 (5)	
H35A	0.8100	0.0534	0.9352	0.049*	
H35B	0.8803	−0.0470	0.9785	0.049*	
H35C	0.9190	0.0793	0.9669	0.049*	
C36	0.76088 (13)	0.27986 (18)	0.95627 (8)	0.0259 (4)	
C37	0.56916 (15)	−0.0520 (2)	0.88233 (10)	0.0367 (5)	
C38	0.5741 (3)	−0.1711 (2)	0.91295 (15)	0.0637 (8)	
H38A	0.5273	−0.2285	0.8861	0.076*	
H38B	0.5628	−0.1547	0.9454	0.076*	
H38C	0.6356	−0.2077	0.9273	0.076*	
C39	0.6996 (2)	0.7183 (3)	0.81098 (13)	0.0624 (8)	
H39A	0.7033	0.7619	0.7793	0.075*	
H39B	0.7155	0.6307	0.8097	0.075*	
C40	0.7703 (3)	0.7727 (4)	0.86721 (17)	0.0810 (11)	
H40A	0.8319	0.7648	0.8707	0.097*	
H40B	0.7687	0.7287	0.8990	0.097*	
H40C	0.7563	0.8602	0.8686	0.097*	

### Atomic displacement parameters (Å^2^)

**Table:**

	*U*^11^	*U*^22^	*U*^33^	*U*^12^	*U*^13^	*U*^23^
Mn1	0.02138 (16)	0.01881 (16)	0.02466 (16)	0.00141 (10)	0.00919 (12)	0.00055 (10)
O1	0.0238 (7)	0.0230 (7)	0.0456 (8)	0.0015 (5)	0.0137 (6)	0.0040 (6)
O2	0.0223 (6)	0.0251 (7)	0.0287 (7)	0.0014 (5)	0.0087 (5)	−0.0004 (5)
O3	0.0329 (7)	0.0229 (7)	0.0430 (8)	0.0018 (6)	0.0186 (6)	0.0032 (6)
O4	0.0725 (12)	0.0277 (8)	0.0485 (9)	0.0103 (8)	0.0294 (9)	0.0003 (7)
O5	0.0375 (8)	0.0311 (8)	0.0272 (7)	0.0049 (6)	0.0122 (6)	−0.0005 (6)
O6	0.0423 (9)	0.0312 (8)	0.0453 (9)	−0.0011 (7)	0.0145 (7)	−0.0087 (7)
N1	0.0218 (7)	0.0212 (8)	0.0254 (7)	0.0026 (6)	0.0079 (6)	0.0025 (6)
N2	0.0298 (8)	0.0310 (9)	0.0271 (8)	0.0025 (7)	0.0148 (7)	0.0013 (6)
C1	0.0238 (9)	0.0236 (9)	0.0285 (9)	0.0016 (7)	0.0084 (7)	−0.0038 (7)
C2	0.0254 (9)	0.0281 (10)	0.0402 (11)	−0.0011 (8)	0.0128 (8)	−0.0081 (8)
C3	0.0305 (11)	0.0302 (11)	0.0725 (16)	−0.0047 (9)	0.0257 (11)	−0.0028 (11)
C4	0.0577 (16)	0.0526 (16)	0.0736 (19)	−0.0074 (13)	0.0397 (15)	0.0136 (14)
C5	0.0496 (15)	0.0335 (13)	0.093 (2)	−0.0084 (11)	0.0355 (15)	−0.0092 (13)
C6	0.0382 (14)	0.0462 (15)	0.116 (3)	−0.0040 (12)	0.0415 (16)	0.0043 (16)
C7	0.0236 (9)	0.0340 (11)	0.0388 (11)	0.0027 (8)	0.0099 (8)	−0.0072 (9)
C8	0.0282 (9)	0.0357 (11)	0.0250 (9)	0.0094 (8)	0.0056 (8)	−0.0043 (8)
C9	0.0305 (10)	0.0400 (12)	0.0339 (10)	0.0135 (9)	0.0049 (8)	0.0011 (9)
C10	0.0385 (13)	0.073 (2)	0.0539 (15)	0.0227 (13)	0.0158 (11)	0.0095 (14)
C11	0.0445 (13)	0.0565 (16)	0.0406 (12)	0.0159 (12)	0.0112 (11)	0.0107 (11)
C12	0.0608 (18)	0.0446 (16)	0.0658 (19)	0.0244 (14)	−0.0077 (15)	−0.0124 (14)
C13	0.0312 (10)	0.0279 (10)	0.0243 (8)	0.0059 (8)	0.0085 (8)	0.0011 (7)
C14	0.0246 (9)	0.0262 (9)	0.0235 (8)	0.0019 (7)	0.0076 (7)	−0.0022 (7)
C15	0.0250 (9)	0.0236 (9)	0.0263 (9)	0.0029 (7)	0.0076 (7)	0.0053 (7)
C16	0.0301 (9)	0.0223 (9)	0.0298 (9)	0.0017 (7)	0.0096 (8)	−0.0042 (7)
C17	0.0245 (9)	0.0316 (10)	0.0291 (9)	0.0027 (8)	0.0104 (7)	−0.0043 (8)
C18	0.0379 (11)	0.0448 (13)	0.0345 (11)	0.0052 (10)	0.0120 (9)	−0.0106 (10)
C19	0.0436 (13)	0.0687 (18)	0.0267 (10)	0.0108 (12)	0.0134 (9)	−0.0070 (10)
C20	0.0446 (13)	0.0624 (16)	0.0304 (11)	0.0108 (11)	0.0202 (10)	0.0081 (10)
C21	0.0384 (11)	0.0418 (12)	0.0339 (11)	0.0039 (9)	0.0206 (9)	0.0070 (9)
C22	0.0236 (9)	0.0281 (10)	0.0359 (10)	−0.0003 (7)	0.0105 (8)	0.0082 (8)
C23	0.0213 (8)	0.0282 (10)	0.0332 (10)	−0.0005 (7)	0.0100 (8)	0.0026 (8)
C24	0.0251 (9)	0.0271 (10)	0.0446 (11)	−0.0018 (8)	0.0129 (9)	0.0012 (9)
C25	0.0257 (9)	0.0366 (11)	0.0373 (10)	−0.0043 (8)	0.0121 (8)	−0.0064 (9)
C26	0.0315 (11)	0.0415 (13)	0.0470 (13)	−0.0067 (9)	0.0099 (10)	−0.0123 (10)
C27	0.0541 (16)	0.0422 (15)	0.0723 (19)	−0.0112 (13)	0.0113 (14)	−0.0152 (14)
C28	0.0325 (12)	0.070 (2)	0.083 (2)	−0.0160 (13)	0.0170 (13)	−0.0335 (17)
C29	0.0725 (19)	0.0625 (19)	0.0508 (15)	−0.0115 (16)	0.0223 (14)	−0.0231 (14)
C30	0.0249 (9)	0.0385 (11)	0.0297 (9)	0.0011 (8)	0.0089 (8)	−0.0005 (8)
C31	0.0223 (9)	0.0333 (10)	0.0276 (9)	0.0034 (8)	0.0107 (8)	0.0026 (8)
C32	0.0273 (9)	0.0321 (11)	0.0312 (10)	0.0050 (8)	0.0093 (8)	0.0048 (8)
C33	0.0372 (12)	0.0412 (13)	0.0396 (12)	0.0103 (10)	0.0060 (10)	0.0066 (10)
C34	0.0391 (12)	0.0374 (12)	0.0503 (13)	0.0061 (10)	0.0198 (10)	0.0167 (10)
C35	0.0376 (11)	0.0367 (12)	0.0422 (12)	0.0145 (9)	0.0149 (10)	−0.0001 (9)
C36	0.0200 (8)	0.0288 (10)	0.0271 (9)	0.0009 (7)	0.0099 (7)	0.0003 (7)
C37	0.0367 (11)	0.0229 (10)	0.0462 (12)	0.0007 (8)	0.0165 (10)	0.0005 (8)
C38	0.094 (2)	0.0286 (13)	0.084 (2)	0.0047 (14)	0.0548 (19)	0.0088 (13)
C39	0.0674 (19)	0.070 (2)	0.0544 (16)	0.0051 (15)	0.0331 (15)	0.0131 (14)
C40	0.062 (2)	0.079 (3)	0.080 (2)	−0.0136 (18)	0.0171 (18)	0.0095 (19)

### Geometric parameters (Å, °)

**Table:**

Mn1—O1	1.8532 (14)	C16—H16A	0.9900
Mn1—O2	1.8770 (14)	C16—H16B	0.9900
Mn1—O3	1.9958 (15)	C17—C18	1.389 (3)
Mn1—N1	2.1058 (16)	C18—C19	1.387 (4)
Mn1—O5	2.2434 (15)	C18—H18	0.9500
Mn1—N2	2.2543 (17)	C19—C20	1.379 (4)
O1—C1	1.337 (2)	C19—H19	0.9500
O2—C36	1.345 (2)	C20—C21	1.382 (3)
O3—C37	1.266 (3)	C20—H20	0.9500
O4—C37	1.253 (3)	C21—H21	0.9500
O5—H41A	0.9215	C22—C23	1.500 (3)
O5—H41B	0.9263	C22—H22A	0.9900
O6—C39	1.412 (4)	C22—H22B	0.9900
O6—H42	0.9153	C23—C24	1.395 (3)
N1—C16	1.484 (2)	C23—C36	1.400 (3)
N1—C22	1.504 (2)	C24—C25	1.388 (3)
N1—C15	1.506 (2)	C24—H24	0.9500
N2—C17	1.333 (3)	C25—C30	1.397 (3)
N2—C21	1.342 (3)	C25—C26	1.535 (3)
C1—C14	1.407 (3)	C26—C27	1.521 (4)
C1—C2	1.420 (3)	C26—C28	1.532 (3)
C2—C7	1.400 (3)	C26—C29	1.545 (4)
C2—C3	1.550 (3)	C27—H27A	0.9800
C3—C4	1.528 (4)	C27—H27B	0.9800
C3—C5	1.533 (4)	C27—H27C	0.9800
C3—C6	1.541 (3)	C28—H28A	0.9800
C4—H4A	0.9800	C28—H28B	0.9800
C4—H4B	0.9800	C28—H28C	0.9800
C4—H4C	0.9800	C29—H29A	0.9800
C5—H5A	0.9800	C29—H29B	0.9800
C5—H5B	0.9800	C29—H29C	0.9800
C5—H5C	0.9800	C30—C31	1.391 (3)
C6—H6A	0.9800	C30—H30	0.9500
C6—H6B	0.9800	C31—C36	1.422 (3)
C6—H6C	0.9800	C31—C32	1.536 (3)
C7—C8	1.396 (3)	C32—C35	1.530 (3)
C7—H7	0.9500	C32—C33	1.531 (3)
C8—C13	1.394 (3)	C32—C34	1.534 (3)
C8—C9	1.541 (3)	C33—H33A	0.9800
C9—C12	1.507 (4)	C33—H33B	0.9800
C9—C11	1.539 (3)	C33—H33C	0.9800
C9—C10	1.539 (4)	C34—H34A	0.9800
C10—H10A	0.9800	C34—H34B	0.9800
C10—H10B	0.9800	C34—H34C	0.9800
C10—H10C	0.9800	C35—H35A	0.9800
C11—H11A	0.9800	C35—H35B	0.9800
C11—H11B	0.9800	C35—H35C	0.9800
C11—H11C	0.9800	C37—C38	1.506 (3)
C12—H12A	0.9800	C38—H38A	0.9800
C12—H12B	0.9800	C38—H38B	0.9800
C12—H12C	0.9800	C38—H38C	0.9800
C13—C14	1.394 (3)	C39—C40	1.501 (5)
C13—H13	0.9500	C39—H39A	0.9900
C14—C15	1.504 (3)	C39—H39B	0.9900
C15—H15A	0.9900	C40—H40A	0.9800
C15—H15B	0.9900	C40—H40B	0.9800
C16—C17	1.508 (3)	C40—H40C	0.9800
			
O1—Mn1—O2	177.70 (6)	N2—C17—C18	122.0 (2)
O1—Mn1—O3	88.60 (6)	N2—C17—C16	116.18 (17)
O2—Mn1—O3	89.65 (6)	C18—C17—C16	121.6 (2)
O1—Mn1—N1	89.26 (6)	C19—C18—C17	118.5 (2)
O2—Mn1—N1	92.70 (6)	C19—C18—H18	120.7
O3—Mn1—N1	170.77 (6)	C17—C18—H18	120.7
O1—Mn1—O5	90.40 (6)	C20—C19—C18	119.4 (2)
O2—Mn1—O5	88.25 (6)	C20—C19—H19	120.3
O3—Mn1—O5	94.41 (6)	C18—C19—H19	120.3
N1—Mn1—O5	94.59 (6)	C19—C20—C21	118.7 (2)
O1—Mn1—N2	91.08 (7)	C19—C20—H20	120.7
O2—Mn1—N2	90.47 (6)	C21—C20—H20	120.7
O3—Mn1—N2	92.22 (6)	N2—C21—C20	122.2 (2)
N1—Mn1—N2	78.84 (6)	N2—C21—H21	118.9
O5—Mn1—N2	173.24 (6)	C20—C21—H21	118.9
C1—O1—Mn1	134.32 (13)	C23—C22—N1	112.42 (16)
C36—O2—Mn1	124.42 (12)	C23—C22—H22A	109.1
C37—O3—Mn1	128.95 (14)	N1—C22—H22A	109.1
Mn1—O5—H41A	116.2	C23—C22—H22B	109.1
Mn1—O5—H41B	96.1	N1—C22—H22B	109.1
H41A—O5—H41B	98.1	H22A—C22—H22B	107.9
C39—O6—H42	105.3	C24—C23—C36	121.28 (18)
C16—N1—C22	109.90 (15)	C24—C23—C22	121.74 (19)
C16—N1—C15	110.22 (14)	C36—C23—C22	116.98 (18)
C22—N1—C15	107.91 (14)	C25—C24—C23	120.8 (2)
C16—N1—Mn1	111.93 (12)	C25—C24—H24	119.6
C22—N1—Mn1	107.80 (11)	C23—C24—H24	119.6
C15—N1—Mn1	108.97 (11)	C24—C25—C30	116.91 (19)
C17—N2—C21	119.11 (19)	C24—C25—C26	123.2 (2)
C17—N2—Mn1	112.33 (13)	C30—C25—C26	119.9 (2)
C21—N2—Mn1	127.22 (15)	C27—C26—C28	109.2 (2)
O1—C1—C14	121.37 (17)	C27—C26—C25	112.2 (2)
O1—C1—C2	118.80 (18)	C28—C26—C25	110.1 (2)
C14—C1—C2	119.83 (18)	C27—C26—C29	108.1 (2)
C7—C2—C1	117.6 (2)	C28—C26—C29	108.9 (2)
C7—C2—C3	122.53 (19)	C25—C26—C29	108.4 (2)
C1—C2—C3	119.92 (19)	C26—C27—H27A	109.5
C4—C3—C5	109.5 (2)	C26—C27—H27B	109.5
C4—C3—C6	108.1 (2)	H27A—C27—H27B	109.5
C5—C3—C6	106.4 (2)	C26—C27—H27C	109.5
C4—C3—C2	109.9 (2)	H27A—C27—H27C	109.5
C5—C3—C2	111.0 (2)	H27B—C27—H27C	109.5
C6—C3—C2	111.8 (2)	C26—C28—H28A	109.5
C3—C4—H4A	109.5	C26—C28—H28B	109.5
C3—C4—H4B	109.5	H28A—C28—H28B	109.5
H4A—C4—H4B	109.5	C26—C28—H28C	109.5
C3—C4—H4C	109.5	H28A—C28—H28C	109.5
H4A—C4—H4C	109.5	H28B—C28—H28C	109.5
H4B—C4—H4C	109.5	C26—C29—H29A	109.5
C3—C5—H5A	109.5	C26—C29—H29B	109.5
C3—C5—H5B	109.5	H29A—C29—H29B	109.5
H5A—C5—H5B	109.5	C26—C29—H29C	109.5
C3—C5—H5C	109.5	H29A—C29—H29C	109.5
H5A—C5—H5C	109.5	H29B—C29—H29C	109.5
H5B—C5—H5C	109.5	C31—C30—C25	124.75 (19)
C3—C6—H6A	109.5	C31—C30—H30	117.6
C3—C6—H6B	109.5	C25—C30—H30	117.6
H6A—C6—H6B	109.5	C30—C31—C36	116.89 (19)
C3—C6—H6C	109.5	C30—C31—C32	121.61 (17)
H6A—C6—H6C	109.5	C36—C31—C32	121.48 (18)
H6B—C6—H6C	109.5	C35—C32—C33	107.83 (18)
C8—C7—C2	123.54 (19)	C35—C32—C34	109.6 (2)
C8—C7—H7	118.2	C33—C32—C34	107.51 (19)
C2—C7—H7	118.2	C35—C32—C31	109.09 (17)
C13—C8—C7	117.34 (18)	C33—C32—C31	112.17 (18)
C13—C8—C9	119.4 (2)	C34—C32—C31	110.61 (17)
C7—C8—C9	123.19 (19)	C32—C33—H33A	109.5
C12—C9—C11	109.7 (3)	C32—C33—H33B	109.5
C12—C9—C10	109.3 (2)	H33A—C33—H33B	109.5
C11—C9—C10	107.0 (2)	C32—C33—H33C	109.5
C12—C9—C8	110.81 (18)	H33A—C33—H33C	109.5
C11—C9—C8	107.95 (18)	H33B—C33—H33C	109.5
C10—C9—C8	112.0 (2)	C32—C34—H34A	109.5
C9—C10—H10A	109.5	C32—C34—H34B	109.5
C9—C10—H10B	109.5	H34A—C34—H34B	109.5
H10A—C10—H10B	109.5	C32—C34—H34C	109.5
C9—C10—H10C	109.5	H34A—C34—H34C	109.5
H10A—C10—H10C	109.5	H34B—C34—H34C	109.5
H10B—C10—H10C	109.5	C32—C35—H35A	109.5
C9—C11—H11A	109.5	C32—C35—H35B	109.5
C9—C11—H11B	109.5	H35A—C35—H35B	109.5
H11A—C11—H11B	109.5	C32—C35—H35C	109.5
C9—C11—H11C	109.5	H35A—C35—H35C	109.5
H11A—C11—H11C	109.5	H35B—C35—H35C	109.5
H11B—C11—H11C	109.5	O2—C36—C23	118.68 (17)
C9—C12—H12A	109.5	O2—C36—C31	122.21 (18)
C9—C12—H12B	109.5	C23—C36—C31	119.10 (18)
H12A—C12—H12B	109.5	O4—C37—O3	124.5 (2)
C9—C12—H12C	109.5	O4—C37—C38	119.5 (2)
H12A—C12—H12C	109.5	O3—C37—C38	115.9 (2)
H12B—C12—H12C	109.5	C37—C38—H38A	109.5
C14—C13—C8	121.6 (2)	C37—C38—H38B	109.5
C14—C13—H13	119.2	H38A—C38—H38B	109.5
C8—C13—H13	119.2	C37—C38—H38C	109.5
C13—C14—C1	120.08 (18)	H38A—C38—H38C	109.5
C13—C14—C15	118.51 (18)	H38B—C38—H38C	109.5
C1—C14—C15	121.34 (17)	O6—C39—C40	114.5 (3)
C14—C15—N1	113.64 (15)	O6—C39—H39A	108.6
C14—C15—H15A	108.8	C40—C39—H39A	108.6
N1—C15—H15A	108.8	O6—C39—H39B	108.6
C14—C15—H15B	108.8	C40—C39—H39B	108.6
N1—C15—H15B	108.8	H39A—C39—H39B	107.6
H15A—C15—H15B	107.7	C39—C40—H40A	109.5
N1—C16—C17	113.15 (16)	C39—C40—H40B	109.5
N1—C16—H16A	108.9	H40A—C40—H40B	109.5
C17—C16—H16A	108.9	C39—C40—H40C	109.5
N1—C16—H16B	108.9	H40A—C40—H40C	109.5
C17—C16—H16B	108.9	H40B—C40—H40C	109.5
H16A—C16—H16B	107.8		

### Hydrogen-bond geometry (Å, °)

**Table:**

*D*—H···*A*	*D*—H	H···*A*	*D*···*A*	*D*—H···*A*
O5—H41A···O6^i^	0.92	1.91	2.799 (2)	160
O6—H42···O4^ii^	0.91	1.81	2.723 (3)	171
O5—H41B···O4	0.93	1.79	2.677 (2)	160
C4—H4B···O1	0.98	2.36	2.991 (4)	122
C5—H5C···O1	0.98	2.28	2.929 (3)	123
C15—H15B···O5	0.99	2.54	3.202 (3)	124
C34—H34C···O2	0.98	2.45	3.102 (3)	123
C35—H35A···O2	0.98	2.32	3.010 (3)	126

Symmetry codes: (i) −*x*+1, *y*−1/2, −*z*+3/2; (ii) *x*, *y*+1, *z*.
